# Occupational Exposure in the Lombardy Region (Italy) to SARS-CoV-2 Infection: Results from the MUSTANG–OCCUPATION–COVID-19 Study

**DOI:** 10.3390/ijerph18052567

**Published:** 2021-03-04

**Authors:** Paola Della Valle, Marco Fabbri, Fabiana Madotto, Pietro Ferrara, Paolo Cozzolino, Elisabetta Calabretto, Marco Italo D’Orso, Ermanno Longhi, Riccardo Polosa, Michele Augusto Riva, Giampiero Mazzaglia, Carmen Sommese, Lorenzo Giovanni Mantovani, The MUSTANG–OCCUPATION–COVID-19 Study Group

**Affiliations:** 1 Center for Public Health Research, University of Milan Bicocca, 20900 Monza, Italy; p.dellavalle1@campus.unimib.it (P.D.V.); m.fabbri10@campus.unimib.it (M.F.); michele.riva@unimib.it (M.A.R.); giampiero.mazzaglia@unimib.it (G.M.); lorenzo.mantovani@unimib.it (L.G.M.); 2 IRCCS MultiMedica, 20099 Sesto San Giovanni, Italy; paolo.cozzolino@multimedica.it (P.C.); elisabetta.calabretto@multimedica.it (E.C.); ermanno.longhi@multimedica.it (E.L.); carmen.sommese@multimedica.it (C.S.); 3 Consortium for Occupational and Environmental Medicine, Department of Medicine and Surgery, University of Milan–Bicocca, 20900 Monza, Italy; marco.dorso@unimib.it; 4 Center of Excellence for the Acceleration of HArm Reduction (CoEHAR), University of Catania, 95131 Catania, Italy; polosa@unict.it; 5 Department of Clinical and Experimental Medicine, University of Catania, 95131 Catania, Italy

**Keywords:** antibody persistence, COVID-19, occupational exposure, seroprevalence, SARS-CoV-2

## Abstract

Sero-epidemiological surveys are valuable attempts to estimate the circulation of SARS-CoV-2 in general or selected populations. Within this context, a prospective observational study was conducted to estimate the prevalence and persistence of SARS-CoV-2 antibodies in different categories of workers and factors associated with positivity, through the detection of virus-specific immunoglobulin G and M (IgG/IgM) in serum samples. Enrollees were divided in low exposure and medium-high groups on the basis of their work activity. Antibody responders were re-contacted after 3 months for the follow-up. Of 2255 sampled workers, 4.8% tested positive for SARS-CoV-2 IgG/IgM antibodies, with 81.7% to IgG only. Workers who continued to go to their place of work, were healthcare workers, or experienced at least one COVID-19-related symptom were more likely to test positive for SARS-CoV-2 antibodies. SARS-CoV-2 antibodies prevalence was significantly higher in the medium-high risk vs. low-risk group (7.2% vs. 3.0%, *p* < 0.0001). At 3-month follow-up, 81.3% of subjects still had antibody response. This study provided important information of SARS-CoV-2 infection prevalence among workers in northern Italy, where the impact of COVID-19 was particularly intense. The presented surveillance data give a contribution to refine current estimates of the disease burden expected from the SARS-CoV-2.

## 1. Introduction

Italy has rapidly become one among the countries most affected by the novel coronavirus disease 2019 (COVID-19) pandemic, after the detection of the first confirmed case in late February 2020 [1,2]. As for other countries, the rapid spread of severe acute respiratory syndrome-coronavirus 2 (SARS-CoV-2) has posed an unprecedented challenge, with a high number of confirmed cases—reaching more than 2.8 million cases and almost 97,000 deaths by February 25, 2021, an enormous death toll and impactful consequences on the entire healthcare service system [1,2,3,4,5].

Since epidemiological surveillance leaves out a great proportion of infected people, in particular asymptomatic or pauci-symptomatic individuals that remain outside contact tracing measures, seroprevalence screenings currently represent the best attempt to describe the actual circulation of SARS-CoV-2 in general or selected populations [6,7,8,9]. Indeed, sero-epidemiological studies for the detection of antibodies against the virus have been conducted worldwide, with the objectives of identifying the exposed population and gathering information on immunization levels in general populations [6,10,11].

In Italy, the nationwide survey conducted by the National Institute of Statistics (ISTAT) found an anti-SARS-CoV-2 IgG seroprevalence of 2.5%. The analysis revealed a number of infections six times higher than that captured with the epidemiological surveillance, also highlighting that half (51%) of the people who developed any antibodies in response to a SARS-CoV-2 infection lived in Lombardy region, among the areas of the country which majorly suffered from the COVID-19 outbreak during the first epidemic wave (March—May 2020) [11].

Beyond the crude prevalence of SARS-CoV-2 antibodies in Italy, it is important defining the risk factors associated with the positivity at antibody tests. As regards occupational exposure, lockdown restrictions determined different level of risks for general workforce populations, mainly depending on whether the workers were allowed to work from home or continued to reach and share workplaces, as well as if they were in close proximity to members of the public [12]. Occupation-related features were therefore factors associated with the possibility of being protected from or infected with SARS-CoV-2 [13]. For instance, research that surveyed healthcare workers (HCW) detected, as expected, an antibody prevalence higher than the general seroprevalence [11,14,15,16]. However, to the best of knowledge, other categories of workers are excluded from specific screening, even those who continued to work during the lockdown periods—for instance the police forces—and are therefore expected to show a greater prevalence of antibody than general population [12].

Emerging literature is suggesting that occupational exposure to the virus might be independently associated with anti-SARS-CoV-2 positivity and emphasized the importance of further research on the infection prevalence in worker populations and main factors associated with antibody positivity [11,13,14]. Within this context, the need of a complete occupational surveillance for SARS-CoV-2 has been claimed, in order to acquire information on anamnestic analysis of the circumstances in which the infection is acquired, as well as the preventive and protection measure to be implemented in workplace [17].

Therefore, with the goal to fill this gap, the presented study aimed to primarily estimate the prevalence and time-persistence of SARS-CoV-2 antibodies, and to suggest factors associated with positivity among different categories of workers from a densely populated vast geographical area of northern Italy, which was hit hardest in terms of cases and deaths during the first epidemic wave (March—May 2020) [5,18].

## 2. Materials and Methods

### 2.1. Study Design and Population

The MUSTANG–OCCUPATION–COVID-19 (Studio epidemiologico di IRCCS MUltimedica, SesTo SAN Giovanni, per valutare negli OCCUPATI il profilO immuNologico durante l’epidemia di COVID-19) project defines a prospective observational study designed to investigate the prevalence and factors associated with SARS-CoV-2 infection through the detection of the positive rate of virus-specific immunoglobulin G and M (IgG and IgM) in serum samples of workers from institutions of the metropolitan area of Milan (Lombardy region, Italy), between 7 May and 31 October.

Institutions were selected on the basis of their estimable risks of infection exposure before and during the Italian lockdown period (9 March–18 May) and their willingness to participate. For this purpose, they were selected: (i) an academic institution, where workers came into contact with hundreds of people on a daily basis before the introduction of lockdown measures; (ii) press agencies, whose workers continued their work during the epidemic period (both from home and in their place of work); (iii) a law enforcement agency, who performed their duty during the lockdown; (iv) a healthcare facility, where HCWs came in contact with COVID-19 patients.

All workers of the involved institutions were invited to participate in the study. In order to be included in the survey, participants must be aged 18 years or over, be employed in one of the selected institutions, and not being previously tested for SARS-CoV-2 infection.

Subjects who met the following criteria were excluded: patients in quarantine for active disease and those in COVID-19 surveillance post-hospital discharge. The participation was voluntary and surveyees were not offered any incentive for their participation in the study and were informed about their right to withdraw at any time without penalty. Each worker was provided with information about the nature and protocol of the research, and informed that all information gathered would be anonymous and confidentiality would be maintained by omitting any personal identifying information. All participants provided written informed consent at the beginning of the enrollment by reading and signed the consent form. In order to monitor anti-SARS-CoV-2 antibody levels persistence, participants were also asked to give their consent to be recontacted in case of positivity at first serological test after 3 months.

### 2.2. Endpoints

The primary endpoint of this research was to evaluate the blood concentration of SARS-CoV-2 antibodies (IgG and IgM) among workers from the metropolitan area of Milan, in order to determinate the prevalence of subjects with altered immunologic profile due to the infection according to SARS-CoV-2 exposure risk during Italian lockdown period. Secondary outcomes included: (i) the assessment of the prevalence of COVID-19-related symptoms and risk of infection during the outbreak period; (ii) monitoring changes in antibody levels after pre-defined timespan (3 months) from the first sampling.

### 2.3. Data Definitions

Two different groups were defined on the basis of workers’ level of exposure at SARS-CoV-2 during Italian lockdown period. Screened subjects were classified as: 1. medium-high exposure level: (i) subjects who came in contact with COVID-19 patients (confirmed or suspect diagnosis); (ii) subjects who continued their usual work by going to their place of work and coming in contact with more than 10 people on a daily basis; 2. low exposure level: subjects who did not meet the previous criteria.

### 2.4. Sample Size

The sample size was determined before the study initiation and, at that time, no data was available in literature about the prevalence of altered immunologic profile in workers according their activity. For this reason, IRCCS MultiMedica conducted a preliminary analysis on 200 workers and observed that the prevalence of altered immunologic profile (due to SARS-CoV-2 infection) was 10.2% in HCWs and 5.2% in non-HCWs. In order to test the difference between the two independent proportions, we estimated a sample size of 1924 workers from the different involved institutions (418 with medium-high exposure level and 1443 with low exposure level), assuming a type I error of 5%, a statistical power of 95% and an allocation ratio between groups of 0.33.

### 2.5. Study Procedures

On-site testing points were set up in each selected institution with trained medical doctors and nurses. Here, blood pressure (BP), heart rate (HR), body temperature, and oxygen saturation (SpO2) were recorded for each worker. Volunteers were also interviewed regarding demographics and professional characteristics (gender, age, highest educational qualification, type of work, professional role), health status, smoking habit, history of symptoms compatible with COVID-19 (i.e., fever, severe tiredness, sore throat, cough, shortness of breath, headache, anosmia, ageusia, nausea, vomiting, diarrhea, or any other influenza-like symptom), previous contacts with suspected or confirmed cases (including household contacts), and other risk factors. Subsequently, a specialized nurse obtained a blood sample, collected at the same testing-point access that the vital signs were checked. All collected data were stored in a dedicated database, using a tailored web-form made available from MultiMedica Data Management Unit (Sesto San Giovanni, Milan, Italy), in order to minimize data input errors and allowing a faster linkage with serological test results. Blood samples were analyzed at MultiLab-Centro MultiMedica (Milan, Italy). Those participants who explicitly stated their wish to be contacted in order to be re-tested were called after 3 months by a trained researcher and invited to present at the MultiMedica testing points where a venipuncture was used to obtain a blood sample for antibody testing. Seroprevalence was assessed through TechnoGenetics [TGS] COVID-19 IgG and TGS COVID-19 IgM chemiluminescent immunoassays (CLIA) for the research of SARS-CoV-2 antibodies (Technogenetics SRL, Milano, Italy) in serum samples. Positivity was intended as antibody levels of an index of 1.0 and 11.5 AU/mL, respectively, for IgM and IgG. According to the manufacturer’s recommendations, the TGS COVID-19 IgG test presented sensitivity of 100% (95% CI, 97.8%–99.9%) and specificity of 99.4% (95% CI, 97.6%-99.9%) at 21st days after symptoms onset. Combined sensitivity of TGS COVID-19 IgG and IgM was estimated at 92.9% at ≤ 7 days and 100% ≥ 15 days; specificity at 98.6% [19,20,21].

### 2.6. Statistical Analysis

Descriptive statistics included counts (percentages) for categorical data and mean (and standard deviation, SD) or median (and interquartile range, IQR) for continuous variables, according to the skewness of distribution. The amount of missing data was low (Appendix A, Table A1) and no assumption was made for missing data. Differences between exposure groups in continuous variables were evaluated by the Mann-Whitney U-test or the Student t-test according to normal distribution, while categorical data were compared with the chi-square test or Fisher exact test. The same approach was used to test differences between subjects resulted negative and positive to both SARS-CoV-2 antibodies (IgG or IgM). A multivariable logistic regression model was used to evaluate the association between the altered immunologic profile and exposure level, after adjusting for relevant confounders. In the multivariable model, the relevant confounders (demographic characteristics and behaviors during lockdown period) were identified through a stepwise regression strategy. This approach combines forward and backward selection methods in an iterative procedure (with a significance level of 0.05 both for entry and retention) to select predictors in the final multivariable model. All p-values were two-sided, with values of < 0.05 considered as statistically significant. Statistical analyses were conducted with SAS software, version 9.4 (SAS Institute, Cary, NC, USA).

### 2.7. Ethics

The institutional ethical review board of the IRCCS MultiMedica (Sesto San Giovanni, Milan, Italy) approved the research protocol, survey instrument, and informed consent form (approval number 423.2020).

## 3. Results

**Figure 1:**
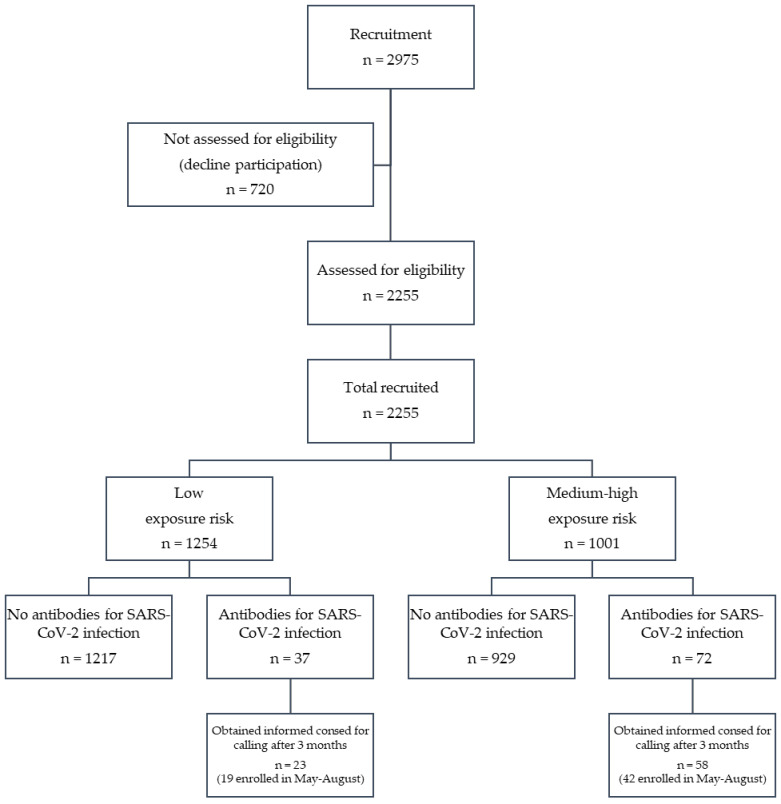
The study flow diagram.

The flowchart of the cohort creation is presented as Figure 1.

A total of 2,255 volunteer workers attended the on-site testing points and were assessed for eligibility. All of them were included in the study, for a total response rate of 75.8%. Complete characteristics of study population are presented in Table 1.

The majority of participants were male (69.6%), with a mean age of 44.5 years and mostly living in Milan province (67.9%). Approximately half the workers were enrolled in May and June (56.0%) and the vast majority were employed as law enforcement (53.8) and office workers (44.0%), while only 2.2% of the sample was constituted by HCWs. Only 37.6% interviewees were allowed to fully work from home by effect of lockdown restrictions, with the remaining workers who kept going to their place of work full-time (40.4%) or part-time (20.1%); another 2.0% completely stopped work activities.

The overall prevalence of SARS-CoV-2 IgG/IgM antibodies was 4.8% and the vast majority (81.7%) of the surveyed workers tested positive to IgG only. Table 2 shows the characteristics of study population according to the positivity at antibody testing.

No differences were found according to sex, age, area of residence, health status, and enrollment period. Those workers who continued to go to places of work, were HCWs, or experienced at least one COVID-19-related symptom were more likely to test positive at SARS-CoV-2 antibodies. Symptoms that were associated with a higher probability of IgM/IgG positivity were fever, sore throat, cold, musculoskeletal pain, or anosmia/ageusia. Current smokers and participants that worked from home during the first months of the outbreak showed a lower prevalence of antibodies.

As regards risk exposure, the low-risk group had a higher probability of testing negative (66.7 vs. 33.3, *p* < 0.001), but the difference disappeared in those enrolled starting from July (49.5 vs. 50.5, *p* 0.37) (Table 2). SARS-CoV-2 antibodies prevalence was significantly higher in the medium-high risk group (7.2% vs. 3.0%, *p* < 0.0001) (Table 3).

Irrespectively of IgG/IgM level, the medium-high risk group self-reported higher probability of having experienced fever, cough, musculoskeletal pain, anosmia/ageusia, and chest pain during the outbreak period than comparators; no other significant difference of self-reported COVID-19-related symptoms was found between groups (Table 3).

The results of the multivariable logistic regression analysis indicated that the probability of testing positive at IgG/IgM antibody was associated to level of exposure at SARS-CoV-2 at work (OR 3.09; 95% CI, 2.03-4.70; *p* < 0.0001), after adjusting for COVID-19-related symptom (OR 5.77; 95% CI, 3.80-8.76; *p* < 0.0001) and age (OR 1.03 per year; 95% CI, 1.01—1.05; *p* < 0.0001) (Table 4).

At 3-month follow-up, 88 subjects enrolled between May and July 2020 with antibodies for SARS-CoV-2 infection resulted to be eligible: of these, 61 had previously given their consent to be re-contacted and 48 performed a second blood sampling for serological test. Antibodies were present in the 81.3% of them (Table 5). Three subjects also tested positive at IgM after 3 months from the first test, being the only antibody type in one worker.

## 4. Discussion

This prospective observational study investigated the prevalence of SARS-CoV-2 infection in the different categories of subjects working in Milan metropolitan area. Evidence before this research suggested the role of work in the risk of infection [11,14,15,16], but this was the first research that documented occupational exposure to SARS-CoV-2 both in HCW and non-HCW workers, allowing to explore strongest predictors of the infection. Furthermore, because the study was designed to obtain information for both low and medium-high groups, significant differences were found according to the type of work and period of enrollment of the participants.

The first important finding is the seroprevalence of IgG/IgM antibodies for the entire sample of 4.8%. The detected proportion of positive subjects was meaningfully higher than that presented in the population-based sero-epidemiological survey conducted by the ISTAT, although remained below the 7.5% of seroprevalence that was detected in Lombardy region in the same survey [11]. There, preliminary data stratified by occupational status found an IgG positivity in the 2.5% for all workers and higher rates in HCWs, in which seroprevalence reached 5.3% and peaked at 9.8% in those working in most hit areas [11]. These disparities in antibody seroprevalences could be attributed to differences in the characteristics of the samples. Again, it should be also assumed that a possible SARS-CoV-2 antigen-specific response disruption in elders due to an impaired adaptive immune response during immunosenescence [22]. Instead, the difference with the regional prevalence (7.5%) was likely attributable to the fact that this value was markedly skewed by the results of Lombardy provinces other than Milan-such as Bergamo and Cremona-where 24% and 19% of population, respectively, tested positive for IgG against SARS-CoV-2 after the first wave of the epidemic [11].

Four out of five MUSTANG–OCCUPATION–COVID-19 participants were employed in essential sectors with close contacts to public, such as law enforcement and healthcare, being therefore mainly exposed at SARS-CoV-2 [12]. Other surveys conducted among HCWs revealed a seroprevalence of antibodies that fell between 4% and 40% [11,16,23,24,25]. This wide variation in the proportion of HCWs who tested positive can be attributed to several reasons, such as the time when the survey was conducted, COVID-19 burden in the area of the healthcare facilities involved, type HCWs enrolled and their exposure risk to SARS-CoV-2 patients, local availability of personal protective equipment [16,23,24,25].

As HCWs, policemen also showed higher reactivity for IgG/IgM antibodies, likely due to duty performance in the areas with high virus exposure and a possible scarce use of personal protective equipment in the first phase of the outbreak. In this regard, no other COVID-19 antibody testing program analyzed SARS-CoV-2 prevalence among police forces, thus it is not possible to compare these results with previous similar findings, highlighting the need of further investigation.

Approximately two-third of the participants who were reactive to the antibodies reported at least one symptom compatible with COVID-19; the most frequent one was fever, mentioned by 67.9% individuals. Having experienced one among possible COVID-19 symptoms was a predictor of SARS-CoV-2 infection, with higher antibody prevalence in those workers who declared fever, sore throat, cold, musculoskeletal pain, and loss of smell and taste. These data documented the presence of a relevant proportion of asymptomatic or pauci-symptomatic subjects that were not detected as cases in the integrated national surveillance [25,26], mostly during the first months of the outbreaks, when swab testing was restricted to symptomatic and severe patients and the capacity of detecting positive cases was low.

By contrast, 32.1% of positive subjects did not experience any symptom, corroborating the presence of an important proportion of healthy asymptomatic patients [6,14]. Examining literature so far available, this proportion ranged from 4% to 41% in similar investigations [6], and it was found to be of 8.6% in Italian adults from general population and thus irrespectively of the occupational status [14].

This sero-epidemiological survey also investigated the presence of possible predictors of SARS-CoV-2 infection. No significant association was found between antibody positivity and subjects’ health status (presence of chronic diseases), assumption of pharmacological therapies, and vaccinations undertaken in the previous 12 months.

Surprisingly, the proportion of current smokers who showed IgG/IgM response was lower than that who tested negative (9.2% vs. 19.6%). As regards smoking habits, the risk of infection by SARS-CoV-2 appeared to be reduced in current smokers also in a large case-control study conducted in Israel, but reasons for these results remained unexplained, although intriguing [27]. Authors suggested possible unique infection mechanisms that might be hindered in smokers, such as an anti-inflammatory mechanism mediated by nicotinic acetylcholine receptor in COVID-19 pathology or angiotensin converting enzyme 2 expression in tissues. On both hypotheses, literature is highly conflicting [28,29]. However, evidence so far available does not allow to conclude that smoking would reduce the risk of SARS-CoV-2 infection [28,29,30]. On the contrary, smoking remains a leading cause of illness and death, and smokers should be encouraged to quit. If potential therapeutic effects of nicotine or nicotinic-cholinergic agonists exists, further in-vitro studies, and observational and clinical research should explore these hypotheses [30].

Compared with previous researches on SARS-CoV-2 antibody prevalence, this survey-in addition to the investigation of occupational exposure to the infection-allowed to study the time evolution of seroprevalence, with differences on the proportion of positive subjects according to the sampling periods. This was particularly true for workers from middle-high risk group, who showed a higher prevalence of antibodies positivity (63.9% vs. 36.1%) before July, namely in first three/four months of the epidemic. The disparity between groups subsequently decreased largely in the second phase, without significant difference, likely due to similar risk of virus exposure across groups after lockdown restrictions (9 March–18 May), as well as social distancing and the use of personal protective equipment that deeply equated the level of protection from SARS-CoV-2 [7,31].

Overall, the results that emerged from this study provided important information on SARS-CoV-2 seroprevalence in a representative sample of workers diversely exposed to the infection. The added value of the MUSTANG–OCCUPATION–COVID-19 study is the possibility to appreciate striking disparities in IgG/IgM positivity by several characteristics of workers and exposure, also highlighting the association of seroprevalence with the measures implemented against the COVID-19 spread. The presence of a great proportion of asymptomatic workers who tested positive confirms the importance of social distancing, the use of personal protective equipment, and the contact tracing measures, as well as their reinforcement.

Further important data on the evolution of antibody immune response during and after the infection emerged from the MUSTANG study. It found that antibodies persisted in the 81.3% of the included individuals after 3 months from the first test. Such a result warrants further follow-up screening of subjects resulting positive in sero-epidemological surveys, through multiple sampling tests—for instance, over 3—12 months after infection —in order to better investigate the duration of humoral immune responses against SARS-CoV-2. In fact, one of the main concerns in creating immunity to SARS-CoV-2 infection by vaccination is whether antibodies persist at least for a period that would allow to neutralize the infection. In these regards, emerging evidence is providing remarkable confirmations that IgG antibodies are maintained in the majority of COVID-19 patients at a mean of 3 months after the onset of symptoms [32,33,34,35].

This study presents a number of strengths. The analyses were conducted using specific and sensitive antibody tests, which strongly correlate with to SARS-CoV-2 infection. The sample was carefully selected and sample size satisfactory, being representative of the general worker population and thus providing reliable estimates of SARS-CoV-2 exposure across participants characteristics. Despite these strengths, some limitations should be acknowledged. First, the survey included workers who voluntarily decided to participate in the study and it should be considered a potential selection bias, where participation could be affected by several factors, such as willingness of reaching on-site testing points and mobility, anxiety, absence of COVID-19-related symptoms. Second, seroprevalence estimates could be affected by geographical distribution of the virus and the research was therefore limited as a real-world study; further research should evaluate immune response in other worker populations. Third, a possible recall bias should be acknowledged regarding the self-reported COVID-19 related symptoms, for this reason we excluded some possible confounders that could have affected the reliability of the data (e.g., duration of symptoms, etc.). Fourth, the low number of HCWs included in the study weakens the generalizability of our findings to this sub-population.

## 5. Conclusions

This study presented SARS-CoV-2 seroprevalence and antibody persistence in a representative sample of workers of a vast geographical area of northern Italy, where the impact of COVID-19 was particularly intense. Here, occupational predictors of infection have been assumed and evaluated, emphasizing the importance of additional protective measures for more vulnerable categories of workers. The research also provided important surveillance data that help to refine current estimates of the disease burden expected from the SARS-CoV-2 spread.

## Figures and Tables

**Table 1:** Characteristics of the study population.

	Total	Low Exposure Risk	Medium-High Exposure Risk	*p*-Value ^
N (%)	2255	1254 (55.61)	1001 (44.39)	-
*Age*, mean ± SD	44.45 ± 9.71	45.65 ± 9.58	42.94 ± 9.67	<0.0001
*Sex, n (%)*				<0.0001
Male	1569 (69.58)	758 (60.45)	811 (81.02)	
Female	686 (30.42)	496 (39.55)	190 (18.98)	
*Enrollment period, n (%)*				
May-June	1263 (56.01)	824 (65.71)	439 (43.86)	<0.0001
July-August	665 (29.29)	327 (26.08)	338 (33.77)	<0.0001
September-October	327 (14.50)	103 (8.21)	224 (22.39)	<0.0001
Municipality*, n (%)				
Province of Milan	1523 (67.90)	852 (68.16)	671 (67.57)	0.7674
Province of Monza-Brianza	277 (12.35)	176 (14.08)	101 (10.17)	0.0052
Other Lombardy provinces	380 (16.94)	191 (15.28)	189 (19.03)	0.0186
Others	63 (2.81)	31 (2.48)	32 (3.22)	0.2904
*Job description, n (%)*				
Healthcare worker	49 (2.17)	6 (0.48)	43 (4.30)	<0.0001
Office worker	993 (44.04)	808 (64.43)	185 (18.48)	<0.0001
Police	1213 (53.79)	440 (35.09)	773 (77.22)	<0.0001
Work type during lockdown period*, n (%)				
Usual workplace	907 (40.36)	151 (12.08)	756 (75.83)	<0.0001
Working from home	845 (37.61)	736 (58.88)	109 (10.93)	<0.0001
Both	451 (20.07)	329 (26.32)	122 (12.24)	<0.0001
Stop of working activity	44 (1.96)	34 (2.72)	10 (1.00)	0.0035
Smoking habit*, n (%)				
Never	1437 (63.90)	805 (64.35)	632 (63.33)	0.6162
Ex-smoker	383 (17.03)	237 (18.94)	146 (14.63)	0.0068
Current smoker	429 (19.08)	209 (16.71)	220 (22.04)	0.0014
*Chronic diseases, n (%°)*				
None	584 (25.90)	870 (69.38)	801 (80.02)	<0.0001
At least one of the following:	1, 71 (74.10)	384 (30.62)	200 (19.98)	
Pulmonary disease, *n* (%°)	79 (13.53)	49 (12.76)	30 (15.00)	0.4527
Cardiac heart disease, *n* (%°)	55 (9.42)	40 (10.42)	15 (7.50)	0.2521
Hypertension, *n* (%°)	260 (44.52)	164 (42.71)	96 (48.00)	0.2221
Renal diseases, *n* (%°)	8 (1.37)	7 (1.82)	1 (0.50)	0.2748
Immunological disorder, *n* (%°)	136 (23.29)	99 (25.78)	37 (18.50)	0.0482
Neoplasm, *n* (%°)	48 (8.22)	35 (9.11)	13 (6.50)	0.2750
Metabolic disorder, *n* (%°)	113 (19.35)	74 (19.27)	39 (19.50)	0.947
Liver disease, *n* (%°)	13 (2.23)	10 (2.60)	3 (1.50)	0.5577
Depression/anxiety, *n* (%°)	70 (11.99)	53 (13.80)	17 (8.50)	0.0612
*Chronic pharmacological therapies, n (%°)*				
None	1642 (72.82)	839 (66.91)	803 (80.22)	<0.0001
At least one	613 (27.18)	415 (33.09)	198 (19.78)	
Aspirin, *n* (%°)	38 (6.20)	25 (6.02)	13 (6.57)	0.7948
Oral anticoagulants, *n* (%°)	11 (1.79)	7 (1.69)	4 (2.02)	0.7529
Antihypertensive, *n* (%°)	246 (40.13)	153 (36.87)	93 (46.97)	0.0170
Statin, *n* (%°)	63 (10.28)	37 (8.92)	26 (13.13)	0.1080
Antidiabetic, *n* (%°)	24 (3.92)	15 (3.61)	9 (4.55)	0.5784
Anticancer, *n* (%°)	10 (1.63)	8 (1.93)	2 (1.01)	0.5128
Cortisone, *n* (%°)	55 (8.97)	36 (8.67)	19 (9.60)	0.7090
Antithyroid agent, *n* (%°)	74 (12.07)	51 (12.29)	23 (11.62)	0.811
Anti-inflammatory agent, *n* (%°)	34 (5.55)	21 (5.06)	13 (6.57)	0.4464
Anxiolytic, *n* (%°)	36 (5.87)	29 (6.99)	7 (3.54)	0.0891
Anti-depressant, *n* (%°)	25 (4.08)	17 (4.10)	8 (4.04)	0.9739
Food supplement, *n* (%°)	167 (27.24)	132 (31.81)	35 (17.68)	0.0002
Immunosuppressive, *n* (%°)	14 (2.28)	13 (3.13)	1 (0.51)	0.0443
*Vaccinations*				
Flu vaccine (Autumn 2019), *n* (%)	234 (10.38)	155 (12.36)	79 (7.89)	0.0005
Pneumococcal vaccine (last 12 months), *n* (%)	12 (0.53)	9 (0.72)	3 (0.30)	0.1752
Other vaccine (last 12 months), *n* (%)	83 (3.68)	53 (4.23)	30 (3.00)	0.1234

^ Comparison between low exposure and medium-high risk exposure groups. * Information on municipality was not available for 12 subjects, work type during lockdown period for 8, smoking for 6. ° Percentage was calculated on subjects with at least one symptom/chronic disease/drug therapy. Sum of percentages was not 100 because each subject could have more than one modality. Abbreviations: SD, standard deviation.

**Table 2:** Characteristics of study population stratified by presence of antibodies for SARS-CoV-2 infection.

	No Antibodies	IgG or IgM Antibodies	*p*-Value ^
N (%)	2146 (95.17)	109 (4.83)	-
Age, mean ± SD	44.37 ± 9.70	46.00 ± 9.80	0.0527
*Sex, n (%)*			0.8046
Male	1492 (69.52)	77 (70.64)	
Female	654 (30.48)	32 (29.36)	
Enrollment period, *n* (%)			
May-June	1202 (56.01)	61 (55.96)	0.2647
July-August	638 (29.73)	27 (24.77)	
September-October	306 (14.26)	21 (19.27)	
SARS-CoV-2 risk exposure			
Low	1217 (56.71)	37 (33.94)	<0.0001
Middle-high	929 (43.29)	72 (66.06)	
Enrollment period and exposure risk			
*May-June*			
Low exposure	802 (66.72)	22 (36.07)	<0.0001
Middle-high exposure	400 (33.28)	39 (63.93)	
*July-August*			
Low exposure	316 (49.53)	11 (40.74)	0.3709
Middle-high exposure	322 (50.47)	16 (59.26)	
*September-October*			
Low exposure	99 (32.35)	4 (19.05)	0.2042
Middle-high exposure	207 (67.65)	17 (80.95)	
Municipality*, *n* (%)			
Province of Milan	1458 (68.26)	65 (60.75)	0.3395
Province of Monza-Brianza	262 (12.27)	15 (14.02)	
Other Lombardy provinces	358 (16.76)	22 (20.56)	
Others	58 (2.72)	5 (4.67)	
Job description, *n* (%)			
Healthcare worker	43 (2.00)	6 (5.50)	0.0285
Office worker	957 (44.59)	36 (33.03)	0.0176
Police	1146 (53.40)	67 (61.47)	0.0994
Work type during lockdown period*, *n* (%)			
Usual workplace	849 (39.71)	58 (53.21)	0.0051
Working from home	818 (38.26)	27 (24.77) ‡	0.0046
Both	432 (20.21)	19 (17.43)	0.4805
Stop of working activity	39 (1.82)	5 (4.59) ‡	0.0590
Smoking habit*, *n* (%)			
Never	1360 (63.55)	77 (70.64)	0.1327
Ex-smoker	361 (16.87)	22 (20.18)	0.3692
Current smoker	419 (19.58)	10 (9.17)	0.0070
Chronic diseases, *n* (%°)			
None	1597 (74.42)	74 (67.89)	0.1292
At least one	549 (25.58)	35 (32.11)	
Pulmonary disease, *n* (%°)	77 (14.03)	2 (5.71)	0.2072
Cardiac heart disease, *n* (%°)	52 (9.47)	3 (8.57)	1.0000
Hypertension, *n* (%°)	242 (44.08)	18 (51.43)	0.3964
Renal disease, *n* (%°)	8 (1.46)	0 (0.00)	1.0000
Immunological disorder, *n* (%°)	129 (23.50)	7 (20.00)	0.6351
Neoplasm, *n* (%°)	45 (8.20)	3 (8.57)	1.0000
Metabolic disorder, *n* (%°)	106 (19.31)	7 (20.00)	0.9199
Liver disease, *n* (%°)	13 (2.37)	0 (0.00)	1.0000
Depression/anxiety, *n* (%°)	66 (12.02)	4 (11.43)	1.0000
Chronic pharmacological therapies, *n* (%)			
None	1567 (73.02)	75 (68.81)	0.3349
At least one	579 (26.98)	34 (31.19)	
Aspirin, *n* (%°)	38 (6.56)	0 (0.00)	0.2585
Oral anticoagulants, *n* (%°)	11 (1.90)	0 (0.00)	1.0000
Antihypertensive, *n* (%°)	231 (39.90)	15 (44.12)	0.6255
Statin, *n* (%°)	58 (10.02)	5 (14.71)	0.3799
Antidiabetic, *n* (%°)	24 (4.15)	0 (0.00)	0.6369
Anticancer, *n* (%°)	8 (1.38)	2 (5.88)	0.1016
Cortisone, *n* (%°)	54 (9.33)	1 (2.94)	0.3501
Antithyroid agent, *n* (%°)	70 (12.09)	4 (11.76)	1.0000
Anti-inflammatory agent, *n* (%°)	32 (5.53)	2 (5.88)	0.7123
Anxiolytic, *n* (%°)	33 (5.70)	3 (8.82)	0.4421
Anti-depressant, *n* (%°)	23 (3.97)	2 (5.88)	0.6429
Flood supplement, *n* (%°)	159 (27.46)	8 (23.53)	0.6168
Immunosuppressive, *n* (%°)	13 (2.25)	1 (2.94)	0.5541
Vaccinations			
Flu vaccine (Autumn 2019), *n* (%)	228 (10.62)	6 (5.50)	0.0873
Pneumococcal vaccine (last 12 months), *n* (%)	12 (0.56)	0 (0.00)	1.0000
Other vaccines (last 12 months), *n* (%)	79 (3.68)	4 (3.67)	1.0000
Clinical parameters			
SpO_2_ (%), median [IQR]	99 [98,99]	99 [98,99]	0.5823
Systolic blood pressure (mmHg), mean ± SD	120.04 ± 13.98	122.54 ± 15.19	0.1045
Diastolic blood pressure (mmHg), mean ± SD	77.62 ± 9.74	78.80 ± 10.60	0.5807
Heart rate (bpm), mean ± SD	76.75 ± 13.34	75.72 ± 11.93	0.4678
Body temperature (°C), mean ± SD	35.97 ± 0.57	36.02 ± 0.55	0.2177
Symptoms COVID-19, *n* (%)			
None	1538 (71.67)	35 (32.11)	<0.0001
At least 1	608 (28.33)	74 (67.89)	
Fever, n(%°)	174 (28.62)	53 (71.62)	<0.0001
Cough, n(%°)	196 (32.24)	26 (35.14)	0.6154
Sore throat/cold, n(%°)	280 (46.05)	24 (32.43)	0.0260
Headache, n(%°)	115 (18.91)	16 (21.62)	0.5767
Muscles/bones/joints pain, n(%°)	107 (17.60)	25 (33.78)	0.0009
Anosmia/ageusia, n(%°)	30 (4.93)	30 (40.54)	<0.0001
Respiratory distress, *n* (%°)	41 (6.74)	6 (8.11)	0.6617
Chest pain, *n* (%°)	26 (4.28)	4 (5.41)	0.5559
Tachycardia, *n* (%°)	15 (2.47)	3 (4.05)	0.4320
Gastrointestinal disorders, *n* (%°)	100 (16.45)	13 (17.57)	0.8067
Conjunctivitis, *n* (%°)	48 (7.89)	5 (6.76)	0.7299
Clinical diagnosis of pneumonia, *n* (%°)	2 (0.33)	5 (6.76)	0.0002

* Information on municipality was not available for 12 subjects, work type during lockdown period for 8, smoking for 6; ^ Comparison between the two groups; ° Percentage was calculated on subjects with at least one symptom/chronic disease/drug therapy. Sum of percentages was not 100 because each subject could have more than one modality; ‡ Those subjects were classified as high risk due to reported contact with COVID-19 confirmed or suspect cases; Abbreviations: IgG: immunoglobulin G; IgM: immunoglobulin M; IQR: interquartile range [1st quartile-3rd quartile]; SD: standard deviation.

**Table 3:** Clinical and serological parameters of study population stratified by SARS-CoV-2 exposure level.

	Total	Low Exposure Risk	Medium-High Exposure Risk	*p*-Value ^
N (%)	2255	1254 (55.61)	1001 (44.39)	-
*Clinical parameters*				
SpO_2_ (%), median [IQR]	99 [98,99]	99 [98,99]	99 [98,99]	0.0648
Systolic blood pressure (mmHg), mean ± SD	120.16 ± 14.05	119.44 ± 14.32	121.07 ± 13.66	0.0079
Diastolic blood pressure (mmHg), mean ± SD	77.68 ± 9.79	77.13 ± 10.27	78.37 ± 9.10	0.0025
Heart rate (bpm), mean ± SD	76.70 ± 13.28	77.63 ± 13.49	75.55 ± 12.93	0.0001
Body temperature (°C), mean ± SD	35.97 ± 0.57	35.91 ± 0.60	36.05 ± 0.52	<0.0001
*Symptoms COVID-19, n (%)*				
None	1573 (69.76)	855 (68.18)	718 (71.73)	0.0685
At least one	682 (30.24)	399 (31.82)	283 (28.27)	
Fever, *n* (%*)	227 (33.28)	103 (25.81)	124 (43.82)	<0.0001
Cough, *n* (%*)	222 (32.55)	116 (29.07)	106 (37.46)	0.0213
Sore throat/cold, *n* (%*)	304 (44.57)	189 (47.37)	115 (40.64)	0.0814
Headache, *n* (%*)	131 (19.21)	86 (21.55)	45 (15.90)	0.0648
Muscles/bones/joints pain, *n* (%*)	132 (19.35)	65 (16.29)	67 (23.67)	0.0162
Anosmia/ageusia, *n* (%*)	60 (8.80)	19 (4.76)	41 (14.49)	<.0001
Respiratory distress, *n* (%*)	47 (6.89)	25 (6.27)	22 (7.77)	0.4436
Chest pain, *n* (%*)	30 (4.40)	11 (2.76)	19 (6.71)	0.0130
Tachycardia, *n* (%*)	18 (2.64)	8 (2.01)	10 (3.53)	0.2198
Gastrointestinal disorders, *n* (%*)	113 (16.57)	67 (16.79)	46 (16.25)	0.8524
Conjunctivitis, *n* (%*)	53 (7.77)	33 (8.27)	20 (7.07)	0.5630
Clinical diagnosis of Pneumonia, *n* (%*)	7 (1.03)	2 (0.50)	5 (1.77)	0.1332
*Antibody test for SARS-CoV-2 infection*				
No antibodies	2146 (95.17)	1,217 (97.05)	929 (92.81)	<0.0001
IgG or IgM antibodies	109 (4.83)	37 (2.95)	72 (7.19)	
Only IgG antibodies	89 (81.65)	31 (83.78)	58 (80.56)	0.8583
Only IgM antibodies	12 (11.01)	3 (8.11)	9 (12.50)	
IgG and IgM antibodies	8 (7.34)	3 (8.11)	5 (6.94)	

^ Comparison between low exposure and medium-high risk exposure groups. *Percentage was calculated on subjects with at least one symptom. Sum of percentages was not 100 because subject could have more than one modality. Abbreviations: IgG: immunoglobulin G; IgM: immunoglobulin M; IRQ: interquartile range [1st quartile-3rd quartile]; SD: standard deviation.

**Table 4:** Relationship between presence of antibody for SARS-CoV-2 infection (IgG/IgM) and characteristics evaluated during lockdown period.

	Adjusted OR (95% CI)	*p*-Value
Multivariable model* on 2,255 subjects (100%)		
Exposure risk (ref. low-risk group)	3.088 (2.027−4.704)	<0.0001
COVID-19 symptoms (ref. No)	5.769 (3.799−8.763)	<0.0001
Age (continuous, in year)	1.026 (1.005−1.048)	0.0132

Abbreviations: OR: odds ratio; CI: confidence interval; Ref: reference category. * In the model, confounders (COVID-19 symptoms, age) were identified through a stepwise regression strategy (significance level of 0.05 both for entry and retent).

**Table 5:** Antibody test for SARS-CoV-2 infection in subjects with altered immunological profile, peformed after 3 months from enrollment.

	Total	Low Exposure Risk	Medium-High Exposure Risk	*p*-Value ^
Patients with serological test after 3 months	48	16	32	-
*Antibody test for SARS-CoV-2 infection*				1.0000
No antibodies	9 (18.7)	3 (18.7)	6 (18.7)	
IgG or IgM antibodies	39 (81.3)	13 (81.3)	26 (81.3)	
Only IgG antibodies	36 (92.3)	12 (92.3)	24 (92.3)	
Only IgM antibodies	1 (7.7)	1 (7.7)	0 (0.00)	0.4073
IgG and IgM antibodies	2 (5.1)	0 (0.0)	2 (7.7)	

^ Comparison between low exposure and medium-high risk exposure groups.

## Data Availability

The data presented in this study are available on request from the corresponding authors. The data are not publicly available due to privacy reason.

## Appendix A

**Table A1:** Missing data in the study population.

	Study Population N = 2255
Age, *n* (%)	0 (0.00)
Sex (male), *n* (%)	0 (0.00)
Enrollment period, *n* (%)	0 (0.00)
SARS-CoV-2 risk exposure, *n* (%)	0 (0.00)
Municipality, *n* (%)	12 (0.5)
Job description, *n* (%)	0 (0.00)
Work type during lockdown period, *n* (%)	8 (0.4)
Smoking habit, *n* (%)	6 (0.3)
Chronic diseases, *n* (%)	0 (0.00)
Chronic pharmacological therapies, *n* (%)	0 (0.00)
Vaccinations	0 (0.00)
*Clinical parameters, n %)*	
SpO_2_ (%)	31 (1.4%)
Systolic blood pressure	24 (1.1%)
Diastolic blood pressure	22 (1.0%)
Heart rate	12 (0.5%)
Body temperature	16 (0.7%)
Symptoms COVID-19, *n* (%)	0 (0.00)
*Antibody test for SARS-CoV-2 infection, n (%)*	
IgG	0 (0.00)
IgM	0 (0.00)
